# Refractory chronic lymphocytic leukemia – new therapeutic strategies

**DOI:** 10.18632/oncotarget.184

**Published:** 2010-11-19

**Authors:** Andrea Schnaiter, Stephan Stilgenbauer

**Affiliations:** University of Ulm, Ulm, Germany

**Keywords:** CLL, refractory, genetics, 17p deletion, p53, TP53 mutation

## Abstract

Treatment outcome of chronic lymphocytic leukemia (CLL) has considerably improved since the introduction of fludarabine (F) as part of the standard therapy. Nevertheless, refractoriness to fludarabine occurs in a significant number of patients and is associated with an unfavorable prognosis. Important risk factors are 17p deletion and/or mutation of TP53. For this subgroup the CD52 antibody alemtuzumab (A) presents a new treatment approach and has already been approved. Meanwhile we have to face also refractoriness to alemtuzumab. Importantly, the monoclonal CD20 antibody ofatumumab has now shown efficacy in F and A double-refractory CLL. The next generation CD20 antibody GA-101 is currently compared to rituximab (R) and will possibly be its more potent successor. Further B-cell antigens are targeted by lumiliximab (CD23), TRU-016 (CD37) and blinatumomab (CD19). Apart from monoclonal antibody therapies, a great number of small molecules are examined for the treatment of refractory and relapsed CLL. Most of these agents aim to overcome apoptosis resistance in CLL cells or influence the microenvironment. Typical targets are regulators of the cell cycle and antiapoptotic molecules like the members of the Bcl-2 family. Up to now the most promising agents appear to be flavopiridol and lenalidomide among others.

## INTRODUCTION

**Figure 1:**
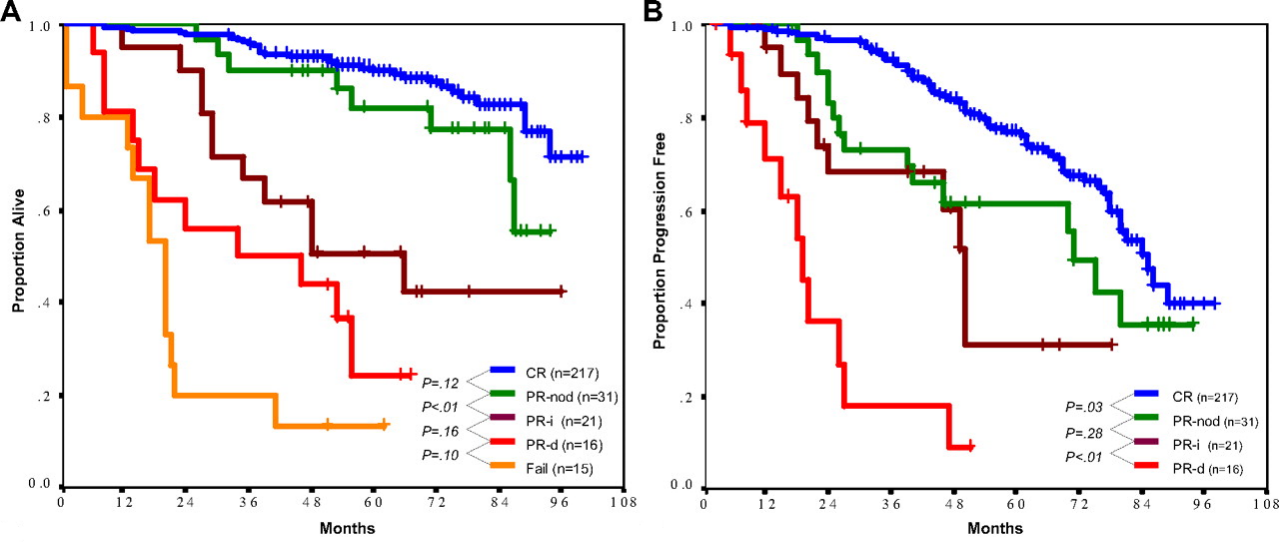
FCR as first-line treatment in CLL at M. D. Anderson Cancer Center.[1,45] A) Overall survival after response. B) Time to progression after response

Chronic lymphocytic leukemia (CLL) is the most common type of leukemia in the Western world and still incurable. What we consider today as gold standard for first line treatment of patients without relevant comorbidities – the anti-CD20 monoclonal antibody rituximab combined with fludarabine and cyclophosphamide (FCR) (Fig. 1) [1] – is often sufficient to achieve durable remission. However we face serious problems if this therapeutic regimen fails and patients turn out to be refractory to fludarabine. In this review we present new strategies beyond conventional chemotherapies which are currently under development or have already entered clinical trials.

**Figure 2:**
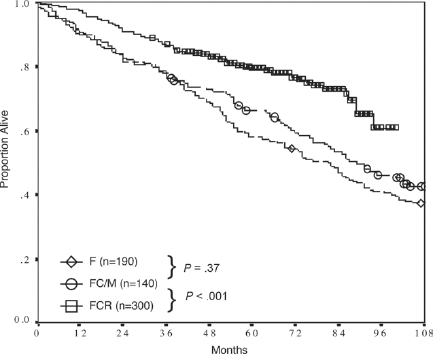
OS in different first line treatment strategies [1] Historical comparison of overall survival for fludarabine (F), fludarabine and cyclophosphamid or mitoxantrone (FC/M) and FCR as CLL first line treatment at M. D. Anderson Cancer Center.

Data of the German CLL Study Group's CLL8 trial showed the advantage of the immuno-chemotherapeutic regimen FCR compared to the former standard FC [2]. 817 patients were included. FCR treatment resulted in a significantly higher complete remission rate (44 % vs. 22 %, p<.001), a significantly longer progression free survival (PFS) (44.7% vs. 64.9% at 3 years, p<.001), and overall survival (OS) (82.5% vs. 87.2% at 3 years, p=.01). It was the first time that a 1^st^ line therapeutic choice in CLL showed significantly prolonged OS in a randomized trial and confirmed previous data in historical comparison performed by MD Anderson Cancer Center investigators (Fig. 2) [2].

## FLUDARABINE REFRACTORINESS AND RELAPSE

**Figure 3:**
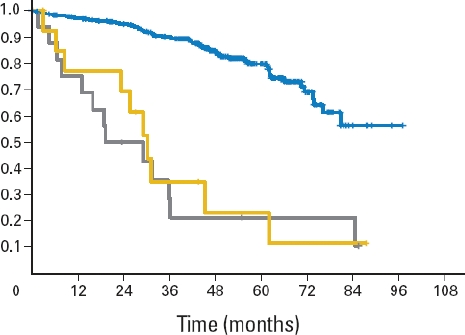
Overall survival (OS) of genetic subgroups in the CLL4 trial (F vs. FC) of the German CLLSG (both treatment arms combined). [7] OS of the group with 17p13 deletion (n=16; grey), sole TP53 mutation (without 17p13 deletion) (n=14; yellow) and the remaining patients (n=277; blue). Median OS was significantly shorter for patients with 17p13 deletion (19.2 months) and sole TP53 mutation (30.2 months) than for patients without either abnormality (not reached; P < 0.001).

Since more than 20 years fludarabine (F) has proven to be effective in the treatment of CLL. In first line monotherapy, there have been response rates of 63 to 80 % [3,4]. Nevertheless not all the patients respond. Genetic analyses revealed the association of fludarabine refractoriness with alterations of the short arm of chromosome 17: 17p-deletion with mutation of the remaining allele of TP53 or mutation of this tumor suppressor gene without deletion. 6 % of untreated patients show alterations in 17p but 53 % of the patients who were refractory to fludarabine [5,6]. A recent analysis of 328 cases of the CLL4 trial of the GCLLSG identified the deletion of TP53 as the strongest prognostic factor for PFS and OS (Fig. 3). Furthermore, deletion of TP53 predicts for non-response to purine analogue based therapy [7,8].

Refractory CLL is defined as no CR/PR or progression within 6 months of the last treatment time point, while relapse of CLL is defined as recurrence of the disease when the patient has reached at least a partial remission for 6 months after therapy. Relapse within 2 or 3 years or refractoriness to F-based therapy influences the prognosis in an unfavorable way [1].New therapeutic strategies beyond conventional regimens like targeted therapy aim to enhance response and survival rates also in the refractory or relapsed situation.

## MONOCLONAL ANTIBODIES

### Rituximab

Because of the promising results of the MD Anderson Cancer Center and the CLL8 trial, rituximab is today part of the CLL gold standard treatment FCR. The monoclonal IgG1-antibody is directed against the CD20 antigen on B lymphocytes. Although CD20 expression levels on CLL cells is much lower than in other B cell lymphomas it has shown its high efficiency in CLL treatment especially in combination treatments.

A phase II study of rituximab monotherapy showed low clinical activity and short duration of response. Rituximab was administered weekly at the common dose of 375 mg/m^2^ over four weeks [9]. The response rate in a dose escalation study in pretreated patients (either fludarabine or alkylating agents) was 36 % with only partial remissions. Response correlated with dose, the maximum dose given was 2.25 g/m^2^. The group of other B cell lymphomas showed response in 60 % of the cases [10].

The strength of rituximab is combination therapy. A response rate of 77 % and complete remissions in 15 % were achieved by combination with bendamustine in a randomized trial of the GCLLSG. Even high risk patients with 11q-deletion, trisomy 12 and unmutated IGHV status had benefit. 17p-deleted patients showed lower response rates [11].

FCR is the standard regimen in CLL first line treatment for patients without relevant comorbidity. Its suitability for relapse has been examined in a phase II trial in 2005. 177 patients were included. Complete remissions were achieved in 25 %, partial remissions in 32 % of the cases. Overall response was 73 %. One third of the patients with CR had also molecular remissions with negative PCR results for the IGHV rearrangement of the CLL clone in their bone marrow. 26 % of the patients discontinued therapy because of heavy myelosuppression. Grade 4 neutropenia was seen in 41 % of the cycles and occurred at least once in 66 % of the patients. 16 % of the patients developed sepsis, pneumonia or other infectious complications which required hospitalization [12].

### Alemtuzumab

**Figure 4:**
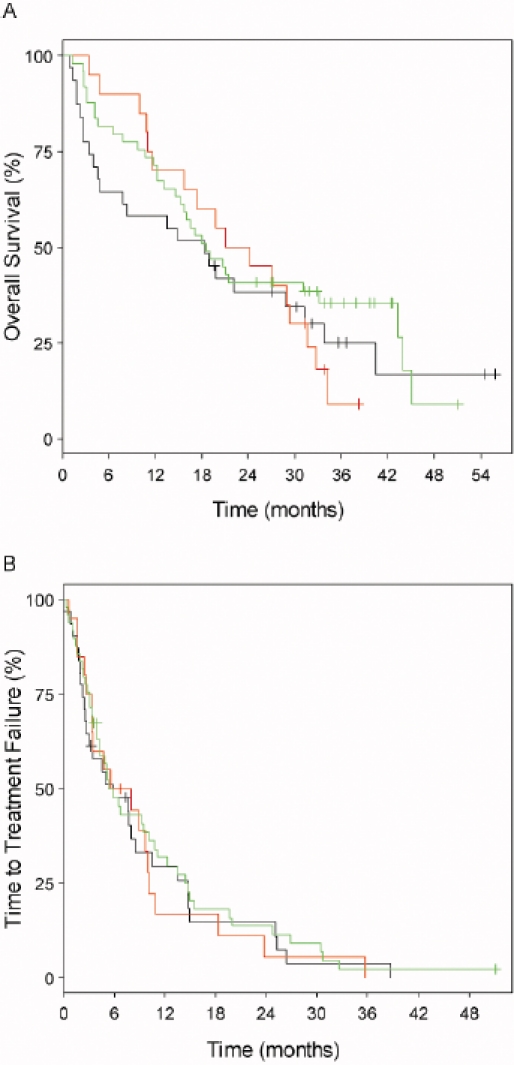
Lack of prognostic impact of genomic aberrations in chemotherapy-refractory chronic lymphocytic leukemia after therapy with alemtuzumab.[14] Genomic subgroups compared were 17p13 deletion (n=31; black graphs), 11q22-q23 deletion (n=20; red graphs) and all other subgroups (n=49; green graphs). A) Median values for overall survival were 18.3, 22.7 and 18.6 months, respectively (log-rank P = 0.661). B) Median values for time to treatment failure were 5.8, 6.8 (95% CI, 3.5 to 10.9) and 5.4 months, respectively (log-rank P = 0.842).

Alemtuzumab (Campath-1H) is a monoclonal IgG1-antibody against CD52 on mature lymphocytes. This drug was approved in 2001 for the treatment of fludarabine refractory CLL in the USA and EU. Since 2008 it is also approved for first line treatment of CLL in patients who are expected to not respond to fludarabine which is the case in 17p-deletion or TP53 mutation. A large multicenter trial in the USA and Europe of i. v. alemtuzumab administration showed better survival data for patients with fludarabine refractory CLL compared to historic cohorts. 93 patients were included. CR rate was 2 %, PR was reached in 31 %. Median time to progression was 4.7 months and median overall survival was 16 months. Transient infusion reactions were the most common adverse events, moreover infectious complications of grade 3 and 4 in 27 % of the patients [13]. The CLL2H trial of the GCLLSG showed that subcutaneous alemtuzumab is as effective as intravenous administration in F-refractory CLL. The overall response rate was 34 % with 4 % of complete remissions. Importantly, side effects of infusion reactions were markedly reduced. Efficiency was the same in all genetic subgroups, notably in 17p- and 11q-deletion, TP53 mutation and unmutated IGHV status (Fig. 4) [14].

Patients with coexpression of CD20 and CD52 might benefit from the combination of rituximab and alemtuzumab. This regimen has been examined over four weeks in 32 patients with relapsed or refractory CLL. Doses were once weekly 375 mg/m^2^ rituximab and twice weekly 30 mg alemtuzumab (with dose escalation in the first treatment cycle from 3 mg over 10 mg to 30 mg). Overall response rate was 63 % with 2 complete remissions and 50 % PR. Adverse events were of grade 2 or less [15]. Another trial evaluated this combination in 40 pretreated patients. 64 % of them were fludarabine-refractory. Rituximab was administered at a dose of 375 mg/m^2^ on day 1, then 500 mg/m^2^ weekly over up to 3 cycles. Alemtuzumab was first applied continuously i. v. at a dose of 30 mg/day for 6 days, followed by 30 mg s. c. twice a week from week 2 on. Infection prophylaxis was trimethoprim/sulfamethoxazole and valacyclovir until up to 3 months beyond the completion of therapy. The overall response rate was 53 % with 18 % of complete remissions. The most adverse events were again infusion-related and well manageable. Infections were seen in 28 % of the patients; 15 % of the patients developed CMF infections which were only detected in blood samples without organ manifestation [16].

The combination of fludarabine and alemtuzumab is called FluCAM. In a phase II trial 36 patients were included. A high overall response rate was reached with 83 % (11 CR, 19 PR), but there were remarkable adverse events: fungal pneumonia in two progressive patients and one case of death caused by E. coli sepsis at CMV reactivation [17].

Alemtuzumab has the highest “single-agent” activity in CLL but involves risks in the form of infectious complications like opportunistic infections and viral reactivations (CMV, HSV). Obligatory is the prophylactic administration of cotrimoxazole and acyclovir or similar besides laboratory monitoring of CMV reactivation. Combination of alemtuzumab with chemotherapy and alemtuzumab maintenance approaches remain experimental and should be performed in clinical trials.

### Ofatumumab (HuMax-CD20)

Like rituximab, ofatumumab binds to CD20 on the surface of B lymphocytes but targets a different epitope and showed enhance in vitro complement dependent cytotoxicity (CDC). Opposite to rituximab ofatumumab is completely of human origin whereas rituximab is a human-murine chimeric molecule. Since October 2009 ofatumumab received accelerated approval by the FDA for the treatment of F and A double-refractory CLL. This decision was mainly based on two clinical trials: a multicenter, sequential dose-escalating cohort and activity-estimating trial and a single-arm, fixed-dose, multicenter trial.

33 patients were included in a phase I/II trial to evaluate safety and efficacy of ofatumumab in patients with refractory or relapsing CLL. In three cohorts different dose escalation schemes of ofatumumab were tested over four cycles. The maximum tolerated dose was not reached and all in all ofatumumab was well tolerated. The majority of the adverse events were infusion-related and transient like pyrexia, fatigue, rash, increased sweating. Infections were reported by 51 % of the patients. Most of them were of grade 1 or 2, one was fatal (infectious interstitial lung disease). The cohort with the highest dose (up to 2000 mg) achieved a response rate of 50 %. There were no complete remissions seen [18].

**Figure 5:**
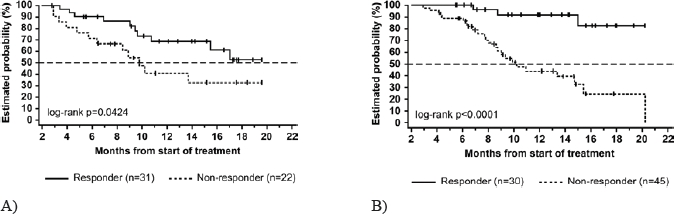
Overall survival in a phase II trial of ofatumumab as single-agent [19] A) Overall survival in fludarabine- and alemtuzumab refractory patients (FA-ref). B) Overall survival in fludarabine-refractory patients with bulky disease (BF-ref)

A phase II trial (NCT00349349) examined ofatumumab as single-agent in patients who were either fludarabine- and alemtuzumab-refractory (FA-ref, n = 59) or fludarabine-refractory and had bulky disease (BF-ref, n = 79) (Fig. 5). Overall response rates (measured during therapy) were 58 % for the FA-ref group and 47 % for the BF-ref group. Former rituximab treatment had no significant influence on efficacy. 54 % of the patients who were pretreated with rituximab responded versus 63 % of the patients who were not exposed (FA-ref group). Furthermore, clinical improvement was achieved for constitutional symptoms, lymphadenopathy, splenomegaly and hematological parameters. Like in the dose-escalating study cited above, the most common adverse events were infusion reactions and infectious complications. Efficacy was lower in CLL with 17p deletion [19].

### Lumiliximab

Lumiliximab is a chimeric monoclonal antibody against CD23. High expression of CD23, a receptor for IgE, is characteristic for CLL cells; on other cells, expression levels are rather low. This fact provides a CLL specific treatment modality.

In a phase I dose-finding trial in 46 CLL patients no remissions were achieved, not even partially, though some clinical activity could be detected. Lymphadenopathy was reduced in 52 % of the patients, in 91 % lymphocyte counts were modestly decreased. Lumiliximab was well tolerated [20].

Based on this knowledge a phase I/II trial was initiated to examine the combination of FCR with lumiliximab in pretreated CLL patients. Inclusion criteria were CD23 positivity, progressive disease according to NCIWG 96. Refractoriness to FCR was exclusion criterium. The overall response rate was 65 %, a complete remission was achieved in 52 % of the patients. Compared to a historic cohort treated with FCR [12], the CR rate was twice as high (52 % vs. 25 %). The toxicity was comparable [21].

### TRU-016

TRU-016 is a SMIP protein (Small Modular ImmunoPharmaceutical) which targets CD37. It is a so called single-chain polypeptide consisting of variable regions (scFv) and the constant regions of human IgG1. CD37 is expressed on normal and transformed B cells, low expression levels are found on T cells, NK cells, monocytes and granulocytes. In in vitro experiments TRU-016 appeared to be more potent than rituximab or alemtuzumab.

In a phase I trial 33 patients with CLL and SLL were included. Applicable doses and two different application schemes were evaluated. TRU-016 was intravenously delivered either once weekly over four cycles or on day 1, 3 and 5 over six weeks in doses of 0.03 mg/kg up to 10 mg/kg. The drug was well tolerated with no unexpected toxicity. Grade 4 neutropenia, herpes zoster and ITP were seen as therapy-related SAEs. At a dose of 3 mg/kg there was detectable biological activity. One patient with 17p deletion achieved a partial remission whereas lymphocytosis decreased about 83% in the median (13 % up to 98 %). There was also an improvement of cytopenia [22].

### GA-101

GA-101 is a humanized and glycoengineered anti-CD20 IgG1 monoclonal type II antibody which binds with high affinity. The resulting antibody-dependent cytotoxicity is 5- to 100-fold greater than that of rituximab. Also direct cytotoxicity is enhanced. CD20 binding leads to homotypic adhesion and following cell death induction is non-apoptotic, actin-dependent and lysosome mediated [23]. Further in vitro studies showed depletion of CLL cells in whole blood assays and the superior efficacy of GA-101 compared to rituximab [24].

In a phase I trial of GA-101 in patients with relapsed or refractory CD20 positive NHL doses between 50 mg and 2000 mg were given on days 1, 8 and 22 every 3 weeks for 9 cycles. 21 pretreated patients were included, 33 % with high risk cytogenetics and 70 % with unmutated IGHV. ORR was 62 % with 1 CRi, 7 PR and 5 SD. GA-101 was well tolerated. Infusion related reactions were the most frequent side effects, followed by hematological toxicity and infectious complications [25].

A phase II study of GA-101 monotherapy in patients with relapsed CD20 positive indolent Non-Hodgkin Lymphoma compares the new CD20 antibody to rituximab and is currently recruiting (NCT00576758). A phase III trial in combination with Chlorambucil in patients with comorbidity is currently active (CLL11 trial of the GCLLSG, NCT01010061).

## SMALL MOLECULES

### Flavopiridol

Flavopiridol is a cyclin-dependent kinase (CDK) inhibitor which targets a broad range of CDKs. CDKs are involved in the regulation of cell cycle, transcription and mRNA processing. In CLL cell lines and primary cells flavopiridol induces apoptosis via activation of caspase-3 [26]. Expression levels of Mcl-1 and XIAP which are constitutively high expressed in CLL cells are decreased [27].

Results of early clinical trials were disappointing [28,29]. However, in vitro studies revealed that protein binding of flavopiridol is much higher with human than with bovine serum. Accordingly, the necessary therapeutic concentration of flavopiridol had been underestimated and a revised schedule was developed. In a phase I trial 52 patients with relapsed, symptomatic CLL or SLL were treated over four weeks once weekly with a loading dose for 30 minutes, followed by 4 hours of continuous infusion. One cycle took six weeks, six cycles were administered. 40 % of the patients achieved a PR, median progression free survival was 12 months. 30 patients suffered acute tumor lysis syndrome, one patient died of it. Most frequent adverse events were neutropenia, diarrhea and cytokine-release syndrome [30]. 121 patients were treated according to the protocol and received infection prophylaxis against HSV/VZV and pneumocystis jirovecii. A total of 43 infectious episodes occurred in 34 patients. Of those 22 were associated with grade 3 or 4 neutropenia. Infectious complications were pneumonia (4 %), bacterial sepsis (1 %), sinusitis or bronchitis (8 %), viral respiratory tract infections (6 %), skin- or soft tissue infections (4 %), catheter associated infections (6 %) and infections of the urinary tract infections (2 %) [31].

In a phase II trial 64 pretreated patients were included. They were treated according to the regimen above with 30 mg/m^2^ by i. v. bolus followed by 30 mg/m^2^ as continuous infusion for the first cycle. If there was no severe tumor lysis syndrome, the dose for continuous infusion was escalated to 50 mg/m^2^ for the second and following cycles. The protocol had to be changed after an amendment: Cycle length was reduced to 4 weeks, the number of treatments per cycle was reduced to 3. 20 mg of dexamethasone were administered to prevent the cytokine release syndrome, ciprofloxacin was added and pegfilgrastim on day 16 of each cycle. 47 % of the patients achieved a partial, 1.6 % a complete remission. The treatment's tolerability was improved. In only 2 % of the cases there was a tumor lysis syndrome [32].

### Lenalidomide

Lenalidomide is an immune-modulating drug, in its structure similar to thalidomide but with more potential in CLL and less side effects. Especially fatigue and neuropathy are reduced. Teratogenicity is expected as it is an analog of thalidomide. In various models, lenalidomide inhibits cytokines like TNFalpha, VEGF and IL-6, stimulates T- and NK-cells, induces apoptosis in tumor cells and inhibits mitosis.

Two phase II trials examined lenalidomide in CLL. A dose of 25 mg p. o. daily was administered for three weeks in a cycle of 28 days. ORR was 47 %. 6 out of 23 fludarabine refractory patients achieved a PR, one patient a CR. Hematological toxicity seems to be a major problem: in 78 % of the cases occurred neutropenia and thrombocytopenia [33]. In the second trial 44 patients were treated continuously with 10 mg daily. Every 28 days the dose could optionally be escalated in steps of 5 mg up to 25 mg daily. ORR was 32 %. 7 % of the patients achieved CR, 10 % PR. 31 % of the patients with 11q- or 17p-deletion (combined) responded, 24 % of the patients with unmutated IGHV and 25 % with fludarabine refractory disease [34]. A subgroup analysis of the trial showed that lenalidomide is also effective in patients with high-risk CLL (combined 11q- or 17p-deletion). ORR in these patients was 38 %, 19 % achieved complete remissions, median progression-free survival was 12.1 months [35], however, more recent data indicate that this is likely due to responses in patients with 11q deletion and there is limited efficacy in cases with 17p deletion.

Typical side effects of lenalidomide treatment are hematological toxicity and at higher doses the tumor flare reaction with lymph node enlargement, leucocytosis, abdominal pain, fever and rashes. Tumor lysis syndrome is also often seen in lenalidomide treatment. Another phase II trial compared the doses already used in the former both trials: 25 mg and 10 mg. Five of 18 patients developed a TLS, two cases were fatal. The protocol was changed to a lower starting dose of 2.5 mg and slow dose escalation which was carefully monitored. Patients got allopurinol and hydration to prevent TLS. Kidney failure requiring dialysis before treatment was an exclusion criterium. TLS has not been observed in the amended protocol [36].

### Oblimersen

Oblimersen is an antisense oligonucleotide which is applied intravenously. It inhibits the translation of the Bcl-2 protein which is a potent apoptosis inhibitor. In CLL expression levels of Bcl-2 are high.

A phase I/II trial examined oblimersen in 40 patients with relapsed or refractory CLL. The maximum tolerated dose was 3 mg/kg/d. Higher doses caused a cytokine release syndrome with or without tumor lysis within 24 to 48 hours. 26 patients were eligible for evaluation. 8 % achieved a partial remission [37].

Another phase II trial in 24 patients, of whom were 19 pretreated, the combination of oblimersen with rituximab and fludarabine was tested. 68 % of the pretreated patients responded, one even achieved a complete remission. Side effects were well manageable. Neutropenia, nausea, fatigue and fever, thrombocytopenia and anemia were the most frequent adverse events [38].

241 patients were included in a phase III trial which compared FC and the combination of oblimersen, fludarabine and cyclophosphamide. 17 % of the patients in the experimental study arm achieved a complete or partial nodular remission at least for 6 months compared to 7 % of the patients treated with FC (p = 0.025). The five-year survival was significantly prolonged in patients who had achieved at least PR. Patients with fludarabine sensitive disease had most benefit [39].

### ABT-263

ABT-263 is a BH3-mimetic which is orally administered. It binds to all members of the family of Bcl-2 proteins with high affinity. In lymphoma cell lines which express high Bcl-2 levels ABT-263 is a potent apoptosis inducer. EC50 is about 1 μM [40]. Primary CLL cells are very sensitive to ABT-737 the precursor drug of ABT-263 which is not orally bioavailable (EC50 = 7 nM, 4 hours incubation time) [41].

A current phase I/IIa trial in patients with relapsed or refractory CLL aims to evaluate pharmacokinetics, safety profile and efficacy of ABT-263. Two application schemes are examined: first 10 to 250 mg of ABT-263 on day 1 to 14 (21-days cycle). This led to pronounced thrombocytopenia as a direct toxic effect on platelets through inhibition of Bcl-XL. To avoid this, patients got a loading dose of 100 mg for one week followed by a continuous application up to 300 mg on all 21 days of the cycle. This reduced fluctuation in the number of thrombocytes as well as early thrombocytopenia. Most frequent adverse events were diarrhea, nausea and vomiting, fatigue, thrombocytopenia (20 %) and neutropenia (12 %). Grade 4 thrombocytopenia was dose-limiting in three patients of the first group, in the second group it was grade 4 thrombocytopenia and nausea also in three patients. These results lead to the recommendation of a loading dose of 100 mg for one week, followed by a continuous dose of 250 mg/d [42].

A phase I trial about the combination of ABT-263 with FCR or BR is currently recruiting, moreover a phase IIb trial of different oral dosage forms (NCT00868413, NCT00918450).

### Obatoclax

The small molecule obatoclax inhibits the binding of the anti-apoptotic members of the Bcl-2 family Bcl-2, Bcl-XL, Bcl-W and Mcl-1 to the proapoptotic proteins Bax and Bak. This might restore the apoptotic machinery of tumor cells.

The EC50 for primary CLL cells is 1.2 ± 0.1 μM. Synergistic effects are seen in combination with fludarabine and chlorambucil [43]. Different doses between 3.5 mg/m^2^ and 14 mg/m^2^ during one hour of infusion and between 20 and 40 mg/m^2^ during 3 hours of infusion were tested for efficacy and safety as well as pharmacokinetic and pharmacodynamics in a phase I trial. One cycle lasted three weeks. The patients were pretreated and either fludarabine refractory or relapsed after a fludarabine containing standard treatment. One patient with bulky disease achieved a partial remission. Remarkable were infusion associated neurological side effects like somnolence, euphoria and ataxia. Hemoglobin levels were increased in three patients which made them independent of blood transfusions. Also their thrombocytopenia had slightly improved [44].

### Histone deacetylase inhibitors (HDAC inhibitors)

The secondary structure of histones is modulated by posttranslational acetylation, methylation and phosphorylation. As a result histones dissociate from the DNA-strand and transcription factors can bind to their promoters. Deacetylation, demethylation and dephosphorylation causes the opposite.

The most well known HDAC inhibitor is valproic acid which is used as anticonvulsant. In primary CLL cells valproic acid induces dose-dependent cytotoxicity via the extrinsic apoptosis pathway. In combination with TRAIL those cells are sensitized for apoptosis. Doses between 0.5 and 5 mM were used [45]. Clinical trials with valproic acid as single-agent (NCT01016990) and in combination with fludarabine (NCT00524667) are recruiting.

Two phase I trials on HDAC inhibitors in CLL have already been published. 10 patients were treated with 13 mg/m^2^ depsipeptide (FK228) in a dose finding study. All the patients were pretreated either with purine analogs or had a contraindication against those drugs. The cycles lasted four weeks, depsipeptide was applied intravenously on day 1, 8 and 15. Treatment was continued for at least two months up to one year. Adverse events were fatigue, nausea and loss of appetite. One patient suffered a tumor lysis syndrome with renal failure and needed hospitalization. There were neither partial nor complete remissions. However biological activity was detected: histone acetylation was increased up to 100 %, p21 expression was augmented [46].

Another phase I trial examined dosage and safety of Belinostat (PXD101). 18 patients with different hematological malignancies were included, two of them had fludarabine refractory CLL. Both were treated for two cycles. One cycle lasted three weeks. At a dose of 600 mg/m^2^ and 900 mg/m^2^ on day 1 to 5 of each cycle they had stable disease. The most frequent side effects were again nausea and vomiting, fatigue and a flush syndrome. Doses up to 1000 mg/m^2^ seem to be well tolerable. There were no distinct responses [47].

### Gossypol/AT-101

AT-101 is an enantiomer of gossypol which binds with high affinity to the BH3 domain of the antiapoptotic members of the Bcl-2 family. At a dose of 20 μM for 24 hours AT-101 induces 72 % apoptosis in the median in primary CLL cells. This was also seen in coculture with a bone marrow stromal cell line [48]. A phase II trial in relapsed or refractory B cell neoplasms (NCT00275431) and a phase II trial in combination with rituximab (NCT00286780) has already been finished. Another phase I/II study about the combination with lenalidomide is still recruiting (NCT01003769).

### Dasatinib

The tyrosine kinase inhibitor dasatinib has shown efficacy in CML and bcr/abl-positive ALL. Dasatinib inhibits c-kit, EGFR and kinases of the Src-family (SFKs). The SFK Lyn is highly expressed in CLL cells and inhibition of SFK activity leads to apoptosis induction in primary CLL cells. Further in vitro experiments showed also synergistic effects of dasatinib with chlorambucil and fludarabine [49]. CLL cells with unmutated IGHV seem to respond better than those with mutation [50].

In a phase II trial in 15 patients a daily dose of 140 mg was administered. All patients had fludarabine pre-treatment, five were refractory. In 2 patients partial remissions were achieved, another 2 had nodal complete remissions, 4 had nodal partial remissions. Adverse events were mainly neutropenia (grade 3 and 4) and thrombocytopenia (grade 3 and 4). All in all dasatinib showed modest activity in CLL [51].

### CONCLUSION

Current biologically-targeted therapeutic strategies against relapsed and refractory CLL include antibody-based therapies as well as small molecules. Rituximab in combination with chemotherapy is very effective in CLL and standard in first-line and second-line therapy. Fludarabine-refractory patients achieve an overall response of 33 % by single-agent alemtuzumab. Today we see patients who are not only fludarabine- but also alemtuzumab-refractory. For those cases ofatumumab is a promising option which has been approved for this situation. Third-generation antibodies like GA-101 show in vitro even more efficacy than rituximab and are currently further evaluated in clinical trials. The most advanced small molecules are today lenalidomide and flavopiridol. Response rates of 20 to 45 % were achieved in relapsed/refractory CLL. A broad range of agents with different mechanisms of action are in development or have already entered clinical trials, but the prognosis of chemotherapy-refractory patients is still unfavorable. Thus new therapeutic strategies are needed and have to prove not only efficacy but also a good safety profile.
